# CD13 is a useful tool in the differential diagnosis of meningiomas with potential biological and prognostic implications

**DOI:** 10.1007/s00428-022-03304-9

**Published:** 2022-02-25

**Authors:** Stefano Marletta, Claudio Luchini, Nicola Sperandio, Evelin Torresani, Alessandro Sorio, Ilaria Girolami, Aldo Scarpa, Albino Eccher, Claudio Ghimenton

**Affiliations:** 1 Department of Pathology and Diagnostics and Public Health, Section of Pathology, University of Verona, Verona, Italy; 2 Department of Pathology and Diagnostics, University of Verona, P.le Stefani n. 1; 37126, Verona, Italy; 3 Division of Pathology, Central Hospital, Bolzano, Italy

**Keywords:** Meningiomas, Meningeal tumors, Immunohistochemistry, Differential diagnosis, CD13

## Abstract

Meningiomas are common tumors of the central nervous system. Although their histological diagnosis is usually straightforward, their differential diagnosis versus other tumors may be challenging at times. The objective of this study is to assess the diagnostic value of CD13 immunoexpression in the differential diagnosis between meningiomas and their morphological mimics. Immunohistochemical analysis for CD13, epithelial membrane antigen, SOX10, and STAT6 was carried out in a large cohort of primary meningeal tumors comprising 225 meningiomas, 15 schwannomas, and 20 solitary fibrous tumor/hemangiopericytomas. Within the meningioma group, the expression of CD13 and epithelial membrane antigen was distinguished in three categories using a semiquantitative score. Most of meningiomas expressed CD13 (94%) and epithelial membrane antigen (96%) while none of the schwannomas nor of the solitary fibrous tumor/hemangiopericytomas was positive for either the two markers. Diffuse positivity for CD13 and epithelial membrane antigen was more common in low-grade meningiomas than in anaplastic ones, which were also more often negative for such markers, especially for CD13 (32%). CD13 is a helpful immunohistochemical marker for the differential diagnosis of meningiomas and their mimics, achieving in combination with epithelial membrane antigen maximal sensitivity (100%) and showing statistically relevant difference of expression in comparison with both schwannomas (*p* < 0.0001) and solitary fibrous tumor/hemangiopericytomas (*p* < 0.0001). Furthermore, loss of CD13 expression could be related to outcome as it is associated with worrisome histological findings, mainly in the setting of anaplastic meningiomas.

**Supplementary Information:**

The online version contains supplementary material available at 10.1007/s00428-022-03304-9.

## Introduction

Meningiomas are among the most common tumors of the central nervous system (CNS) in adults, accounting for 15–30% of primary intracranial tumors and for 25% of intraspinal ones [1]. According to World Health Organization (WHO) criteria, they are classified into fifteen histotypes and into three grades of malignancy, showing increasing recurrence risk: grade I meningiomas (benign) which are the most frequent and show a low risk of recurrence; grade II meningiomas (atypical, chordoid, or clear cell), less common but displaying a higher rate of recurrence; grade III or malignant meningiomas (anaplastic, rhabdoid, and papillary), the rarest ones but associated with severe prognosis and poor overall survival [2].

Meningiomas’ histological features belong to a wide spectrum of different morphological appearances. Albeit they are usually easily recognized on hematoxylin and eosin–stained sections, sometimes reaching the proper diagnosis can be really challenging, as other tumors of the meninges may have a similar histological aspect: spinal cord schwannomas can be really close to meningiomas and meningeal solitary fibrous tumor/hemangiopericytomas (SFT/HPC), although rare, may sometimes closely resemble fibrous and atypical meningiomas as well. In such cases, ancillary tests, like immunohistochemical exams, play a key role in sorting the diagnostic quandary out: meningothelial tumors are usually positive for epithelial membrane antigen (EMA) and progesterone receptors (PR), while schwannomas and SFT/HPCs commonly stain respectively positive for S100 and SOX10 and for CD34 and STAT6. However, S100 and CD34 can be expressed by a significant percentage of meningiomas, especially fibrous ones, and, on the other hand, EMA- and PR-positive meningeal SFT/HPCs have been reported [3]. Furthermore, despite STAT6 expression being widely acknowledged as strongly specific for SFT/HPCs, such marker is not available in all laboratories. Therefore, in such circumstances, assessing the correct diagnosis can be extremely difficult.

CD13, also known as aminopeptidase N (APN), is a Zn-dependent membrane alanyl-aminopeptidase whose biological role has been linked to invasiveness and neoangiogenesis in many human malignant tumors [4]. Lack of expression of CD13 has also been associated with aggressive and high-grade meningiomas [5], but its use in the diagnostic process of meningeal tumors has not been studied yet.

In this study, we tested CD13 expression in a large cohort of meningiomas, schwannomas, and SFT/HPCs and we sought to investigate whether it could be helpful in the differential diagnosis among these tumors. Furthermore, by associating the levels of CD13 with the different grades of meningiomas, we tried to identify any correlation between its expression and gain of aggressive histological features.

## Materials and methods

### Tumor samples

Consecutive samples from 225 meningiomas, 15 schwannomas, and 20 SFT/HPCs were retrieved from the files of the Pathology Department of the University of Verona (Italy). All were primary meningeal tumors. Meningiomas were graded according to the 2016 WHO classification [2] and classified as follows: 100 WHO grade I meningiomas, 100 WHO grade II atypical meningiomas, and 25 WHO grade III anaplastic meningiomas. All slides were reviewed by an experienced neuropathologist (C.G). Grade I and grade II meningiomas were further classified into different subtypes according to their histological characteristics. If morphological features of more than one subtype were present, the tumor was classified considering the histological findings of which subtype was mainly represented.

### Histology and immunohistochemistry

**Fig. 1:**
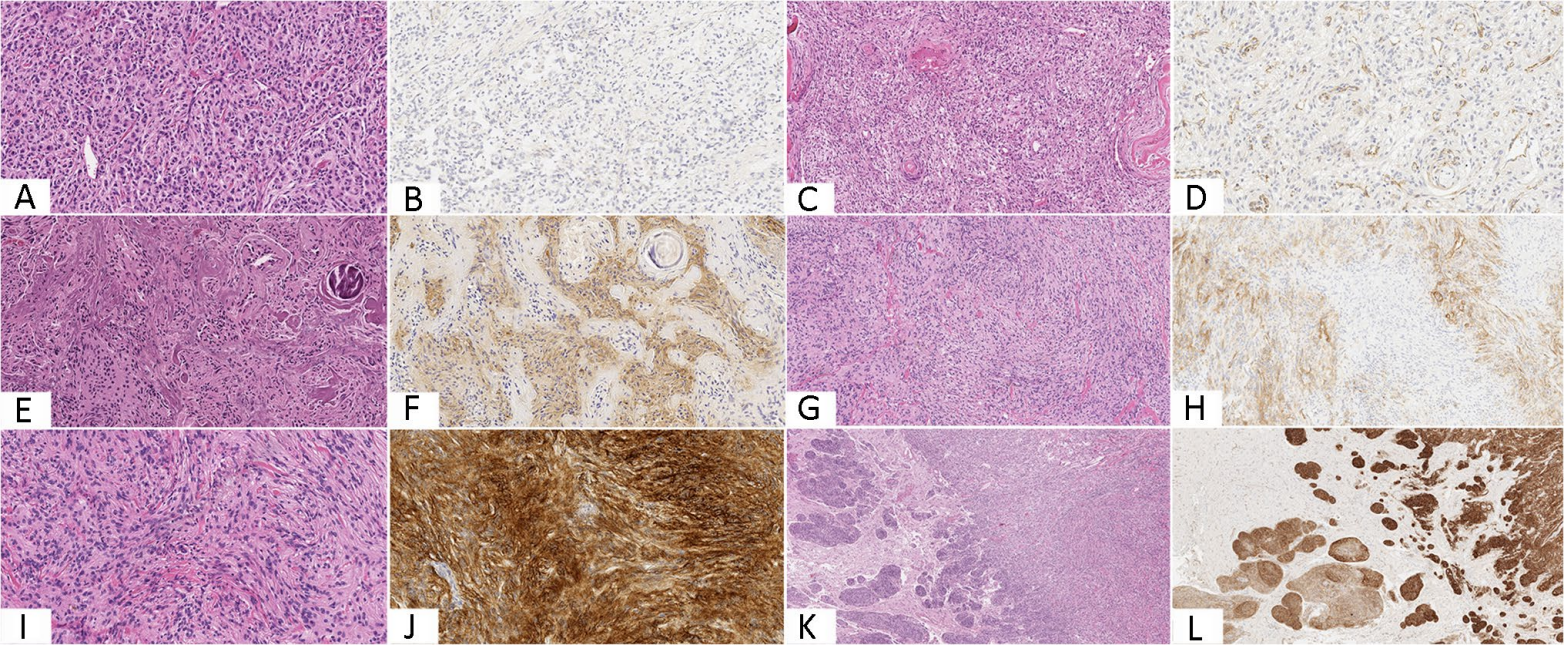
Examples of different scoring considered in the present series: morphological (**A**, **C**) and immunohistochemical appearance of meningiomas labelling negative for EMA (**B**) and CD13, conversely staining endothelial cells (**D**); tumors partially expressing EMA (**E**, **F**) and CD13 (**G**, **H**) revealing groups of positive neoplastic cells along with negative ones; diffuse staining for EMA (**I**, **J**) and CD13 (**K**, **L**). This latter one homogeneously maintained at the front of invasion of the neoplasm within the surrounding brain parenchyma (original magnifications × 200 (**A**, **B**, **D**, **E**, **F**, **I**, **J**), × 100 (**C**, **G**, **H**), and × 50 (**K**, **L**))

Samples obtained were fixed in 10% formalin and embedded in paraffin. Paraffin-embedded tissue blocks were cut into 2- to 3-μm sections and stained using hematoxylin–eosin. Sections from tissue blocks of the tumors studied were immunohistochemically stained with the following antibodies: EMA (clone E29, dilution 1:400; Dako), CD13 (NCL-L-CD13-304, clone 38C12, dilution 1:100; Novocastra), SOX10 (clone BC34, dilution 1:100, Biocare Medical), and STAT6 (clone D1, dilution 1:200; Santa Cruz). All samples were processed using a sensitive Bond Polymer Refine detection system in an automated Bond immunohistochemistry instrument (Leica Biosystems, Germany). As the tumors showed a rather homogenous intensity of expression of the markers tested, immunoexpression of CD13 and EMA in all meningiomas’ samples was evaluated only basing on the percentage of positive cells and semiquantitatively graded as follows: score 0 no expression; score 1 expression in 1–40% of tumor cells; score 2 expression in more than 40% of tumor cells. Examples of each scoring grade for both EMA and CD13 are provided in Fig. 1. Evaluation of at least ten H.P.F was considered reliable for assigning tumors to a specific immunohistochemical score.

### Statistical analysis

The immunohistochemical expression of the previously listed markers was recorded. Namely, meningiomas and their subtypes were tested for CD13 and EMA; schwannomas for CD13, EMA, and SOX10; and SFT/HPCs for CD13, EMA, and STAT6. In order to find out statistically relevant correlations, the exact Fisher test was carried out for comparing the results obtained among the different tumor categories and, for meningiomas, according to the aforementioned three cut-off levels. The results were considered statistically significant if the *p* value was less than 0.05.

## Results

### Meningiomas

**Fig. 2:**
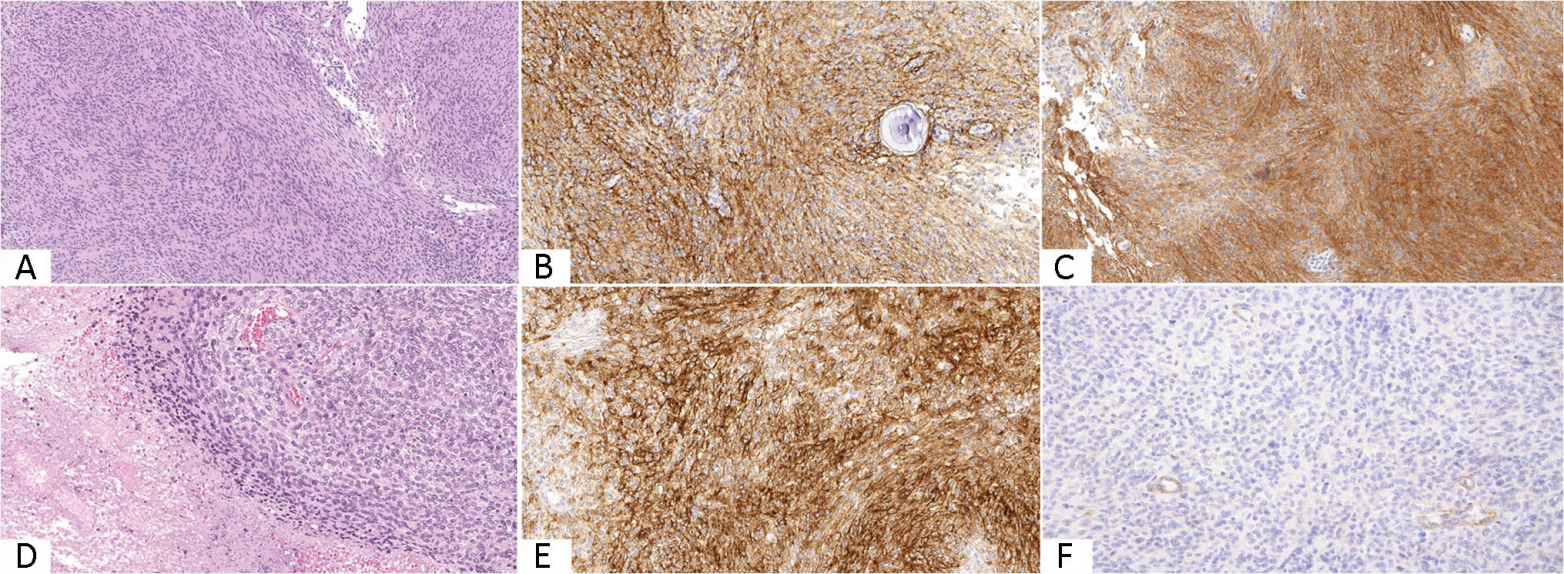
Morphological appearance of grade I (low grade) meningioma (**A**); immunohistochemically staining strongly and diffusely for EMA (**B**) and for CD13 (**C**). Histological features of grade III (high grade) meningioma (**D**) immunolabeling for EMA (**E**) but lacking CD13 expression (**F**) (all pictures were taken at original magnification × 200)

The pathological features of meningiomas are tabulated in Table 1 and shown in Fig. 2, along with their immunohistochemical findings. Among the 225 grade I and grade II meningiomas, 154 were histologically classified as meningothelial, 35 as fibrous, 5 as secretory, 3 as microcystic, 2 as psammomatous, and 1 as clear cell meningiomas respectively. Namely, within the grade I meningiomas category, 69 tumors were diagnosed as meningothelial, 21 as fibrous, 5 as secretory, 3 as microcystic, and 2 as psammomatous. On the other hand, in the grade II subset, 85 atypical meningiomas showed a meningothelial morphology, 14 a fibrous one, and 1 a clear cell appearance.

**Table 1:** Histological features of grade I and grade II meningiomas

Histotype	Grade I + grade II (*n*)	Grade I (*n*)	Grade II (*n*)
Meningothelial	154	69	85
Fibrous	35	21	14
Microcystic	3	3	-
Psammomatous	2	2	-
Secretory	5	5	-
Clear cell	1	-	1
Total	200	100	100

As for immunohistochemistry, EMA expression was respectively detected positive in 96% (216/225) of all meningiomas, 98% (98/100) of grade I meningiomas, 96% (96/100) of grade II meningiomas, and 88% (22/25) of grade III meningiomas. Taken together, grade I and grade II meningiomas stained positive for EMA in 97% (194/200) of the cases. Looking at the detailed meningiomas’ scores, of the overall 216 positive tumors, 31% of them (68/216) revealed weak EMA positivity (score 1) while the remaining 69% (148/216) showed strong positive staining for such marker (score 2). Score 2 was more common in meningioma grade I (71/100), in meningioma grade II (68/100), and in both grades I and II (139/200), than in meningioma grade III (9/25), reaching statistically significant differences (grade I vs. grade III, *p* = 0.0020; grade II vs. grade III, *p* = 0.0052; both grade I + II vs. grade III, *p* = 0.0015). With regard to CD13, it was expressed by 94% (211/225) of all meningiomas, 99% (99/100) of grade I meningiomas, 95% (95/100) of grade II meningiomas, 68% (17/25) of grade III meningiomas. When considering both grades I and grade II meningiomas, CD13 was overall expressed by 97% (194/200) of the tumors. As far as the semiquantitative scoring was concerned, among the 211 CD13-positive meningiomas, 27% of the tumors (56/211) showed weak (score 1) and 73% (155/211) strong (score 2) staining respectively. Similar to EMA, score 2 was more common in meningioma grade I (76/100, 76%), in meningioma grade II (71/100, 71%), and in both grades I and II (147/200, 73%), than in meningioma grade III (8/25, 32%), reaching statistically significant differences (grade I vs. grade III, *p* < 0.0001; grade II vs. grade III, *p* = 0.0005; both grade I + II vs. grade III, *p* < 0.0001).

### Schwannomas and SFT/HPCs

All of the retrieved schwannomas stained positive for SOX10 (15/15), as well as all the SFT/HPCs for STAT6 (20/20). None of the schwannomas nor of the SFT/HPCs of the casuistry tested positive for either EMA or CD13.

Clinical and pathological features of the tumors of the present series are provided in Table S1.

### Diagnostic performance of immunohistochemical markers in the differential diagnosis

**Fig. 3:**
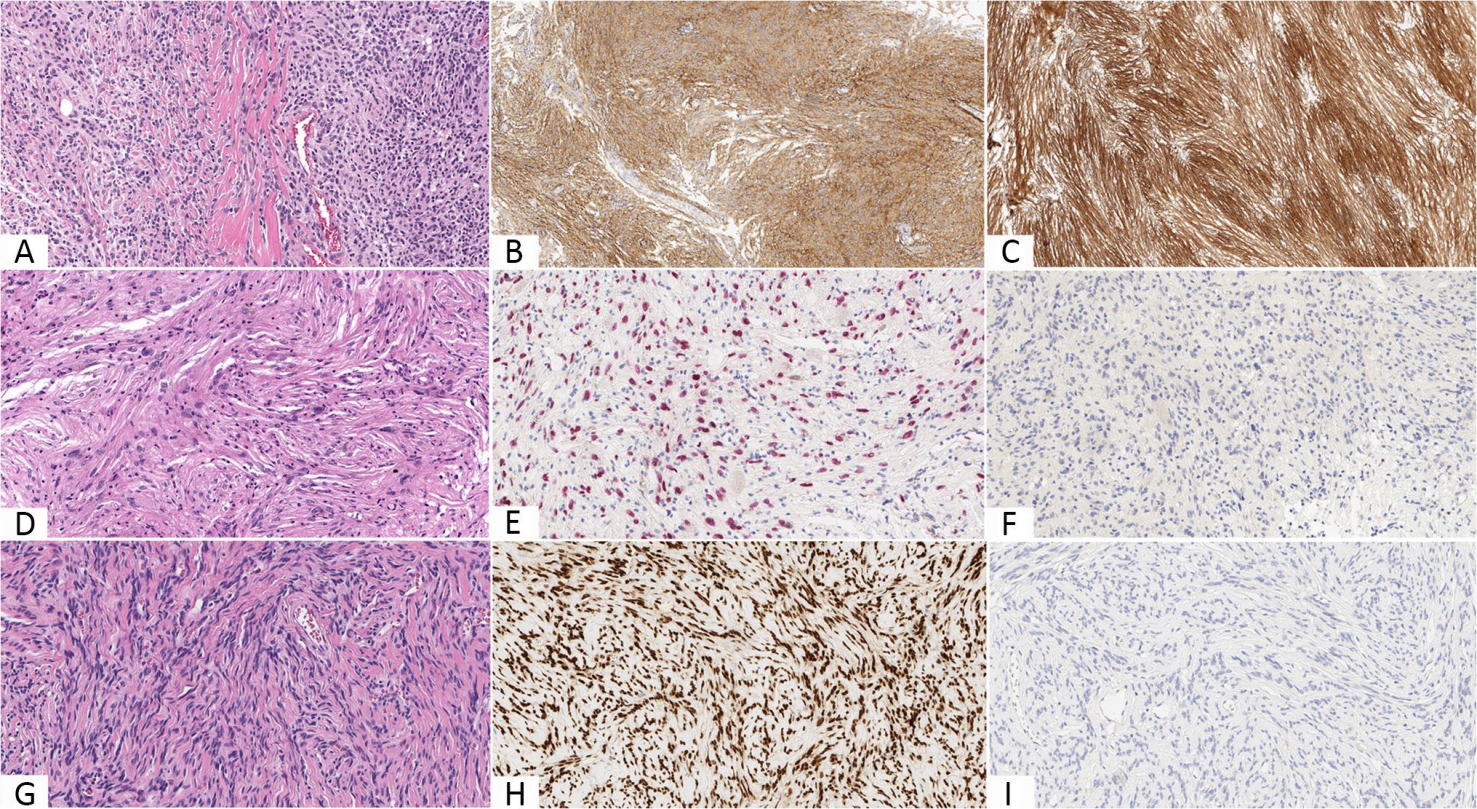
Fibrous meningioma (**A**) immunohistochemically expressing both EMA (**B**) and CD13 (**C**). Schwannoma (**D**) immunolabeling for SOX10 (**E**) but not for CD13 (**F**). Solitary fibrous tumor/hemangiopericytoma (**G**) staining positive for STAT6 (**H**) but negative for CD13 (**I**) (original magnifications × 200 (**A**, **B**, **D**, **E**, **F**, **G**, **H**, **I**) and × 100 (**C**))

The immunohistochemical results of the meningiomas, schwannomas, and SFT/HPCs are listed in Table 2 and a resuming comparison of representative examples is shown in Fig. 3. Using the Fisher exact test, CD13 and EMA expression showed a strong statistically significant correlation with the diagnosis of meningioma in comparison with both schwannomas (*p* < 0.0001) and SFT/HPCs (*p* < 0.0001), regardless of the grade and the histological subtype. It is to note that all negative CD13 tumors (14/225) showed positivity for EMA, as vice versa CD13 expression was observed in all the EMA negative ones (9/225), this latter neoplasms all showing a meningothelial or fibrous morphology. On the other hand, in comparison with both CD13 and EMA, SOX10 expression significantly supported a diagnosis of schwannoma (*p* < 0.0001) as well as STAT6 staining that of SFT/HPC (*p* < 0.0001).

**Table 2:** Immunohistochemical scoring for CD13 and epithelial membrane antigen (EMA)

Tumor	Positive, *n* (%)		Score 1, *n* (%)		Score 2, *n* (%)		Negative, *n* (%)	
	CD13	EMA	CD13	EMA	CD13	EMA	CD13	EMA
All meningiomas	211 (94%)	216 (96%)	56 (25%)	68 (30%)	155 (69%)	148 (66%)	14 (6%)	9 (4%)
Grade I	99 (99%)	98 (98%)	23 (23%)	27 (27%)	76 (76%)	71 (71%)	1 (1%)	2 (2%)
Grade II	95 (95%)	96 (96%)	24 (24%)	28 (28%)	71 (71%)	68 (68%)	5 (5%)	4 (4%)
Grade III	17 (68%)	22 (88%)	9 (36%)	13 (52%)	8 (32%)	9 (36%)	8 (32%)	3 (12%)
Grade I + grade II	194 (97%)	194 (97%)	47 (23%)	55 (27%)	147 (73%)	139 (69%)	6 (3%)	6 (3%)
Grade II + grade III	112 (90%)	118 (94%)	33 (15%)	41 (18%)	79 (63%)	77 (62%)	13 (10%)	7 (6%)
Schwannomas	0 (0%)	0 (0%)	-	-	-	-	15 (100%)	15 (100%)
SFT/HPC	0 (0%)	0 (0%)	-	-	-	-	20 (100%)	20 (100%)

*SFT/HPC* solitary fibrous tumor/hemangiopericytoma, *EMA* epithelial membrane antigen

With regard to meningiomas’ classification, among the different histological subtypes, no statistically significant difference of CD13 and EMA expression was observed.

## Discussion

Meningiomas are among the most common primary CNS tumors and can display a broad range of morphological findings and biological behavior [2]. In the great majority of the cases, the diagnosis is easily assessed on hematoxylin and eosin–stained sections but sometimes they may show overlapping histological features with other CNS tumors, among which schwannomas and SFT/HPCs are the most common ones [6]. Namely, not so rarely fascicles of fibrous meningiomas may display nuclear palisading reminiscing Verocay bodies, quite strikingly resembling schwannomas, especially intraspinal ones. While S100 is a highly sensitive marker for schwannomas, it is far from being specific for such tumors, as it has been found to be expressed in a wide percentage of fibrous meningiomas, in some articles reaching almost half of the whole casuistry [3, 7]. Additionally, as far as SFT/HPCs are concerned, the patternless architecture and interlacing fascicles of solitary fibrous tumors may closely mimic a fibrous meningioma [8]; on the other hand, some highly cellular and mitotically active tumors with a prominent hemangiopericytoma pattern can be misdiagnosed as atypical meningiomas [6]. Distinguishing between these two entities in this latter setting is of paramount relevance as meningeal SFT/HPCs with worrisome histological features carry a much severe clinical behavior than atypical meningiomas, showing a high rate of recurrences, distant metastasis, and cancer-related death [9]. Although immunohistochemistry is usually helpful in allowing physicians to reach the proper diagnosis, in some instances with atypical and unconventional immunophenotypes, it could be very challenging to figure the quandary out. Specifically, even though EMA and PR are considered some of the most reliable and sensitive immunohistochemical markers for meningiomas, they could be negative in a few of them [7] and, above all, they are expressed by a significant percentage of SFT/HPCs [2, 3]. On the other hand, the expression of CD34 can be lost in several SFT/HPCs, especially among those showing malignant features [10], while may be detected in about 8–23% of all meningiomas [3, 7]. In this setting, nuclear STAT6 immunohistochemical positivity, result of the NAB2-STAT6 gene fusion, is a very useful hint in supporting a SFT/HPC diagnosis indeed [11, 12]. Nevertheless, 5–10% of meningeal SFT/HPCs have been found to stain negative for STAT6 [13] and such marker is not nowadays available in all pathology laboratories.

In these scenarios, various immunohistochemical markers have been proposed for helping pathologists to handle the most difficult cases, but even with the most promising ones, the diagnostic problem may sometimes remain unsolved. For instance, somatostatin receptor 2A (SSTR2A) expression has been severally accustomed to meningiomas, reaching high levels of sensitivity and specificity, but it can also be observed in a significant amount of SFT/HPCs and other tumors involving the nervous system, like synovial sarcomas, pPNET, gliosarcomas, and perineuromas [2, 3, 14]–[15].

Aminopeptidase N (APN), also known as CD13, is zinc-dependent metalloenzyme found in many human organs, tissues, and cell types. It is normally involved in antigen presentation and other functions [4] and its expression has been studied in a lot of hematological and solid human malignancies, including breast [16, 17], ovarian [18, 19], colon [20], and thyroid cancer [21]. While the diagnostic utility of CD13 in breast and thyroid has been reported [22], it has never been evaluated for the differential diagnosis of meningeal tumors. In our cohort, we found a high percentage of CD13-positive meningiomas (94%), even higher when considering grade I (99%) and grade II (95%) neoplasms, while none of the schwannomas nor of the SFT/HCPs tested positive for it. As expected, strong expression of EMA was observed as well (96% of all meningiomas). Furthermore, all of the CD13-negative meningiomas were positive for EMA and vice versa, no matter what the histological grade was. Therefore, our results indicate that the combination of these 2 markers facilitates the detection of all meningiomas and that evaluation of CD13 expression could be really helpful in the differential diagnosis with SFT/HCPs and schwannomas. Namely, in a meningeal tumor with ambiguous morphological features and immunophenotype, negative staining for CD13 strongly suggests the diagnosis of SFT/HCP. This could be especially worth it for those institutions that do not dispose of STAT6 and which, therefore, cannot rely on these ancillary assays. Similarly, negative expression of CD13 and EMA combined with SOX10 positivity supports a diagnosis of meningeal schwannoma.

Different experimental studies have investigated the potential role of CD13 in tumor biology and it has been associated with various characteristics of malignant phenotype, such as angiogenesis [23, 24], invasion [21], and metastasis [25]. Moreover, high levels of CD13 have been repeatedly linked to severe prognosis and shorter overall survival [26]–[27]. On the other hand, as for instance observed in gastric cancer [28], in meningiomas, CD13 expression has been found to be negatively correlated with higher tumor grade, brain and dural invasion, and recurrence [5]. This negative correlation between CD13 and aggressive characteristics, contrasting with what demonstrated in many other types of cancer, could be explained by the broad spectrum of biological functions played by this molecule in different organs and tumors: with regard to meningiomas, an aggressive behavior of CD13-negative neoplasms may be at least partially linked to loss of its regulatory role on the function of secreted protein, acidic and rich in cysteine (SPARC, osteonectin, or BM-40) [5], a glycoprotein associated with proliferation, cellular adhesion, and angiogenesis and with meningioma invasiveness, increased grade, recurrence, and reduced survival [29]–[30]. The present work is a pure diagnostic study, not focusing on prognostic aspects. This notwithstanding, our results agree with what previously reported, as CD13 was significantly less expressed in anaplastic meningiomas (68%) than in benign (99%) and atypical ones (95%); furthermore, the positivity detected within the anaplastic meningiomas’ group was much more often of weak type than in the other groups, involving a minority of tumor cells. In the near future, it would be really interesting to demonstrate whether any relevant link exists between CD13 expression and prognostic outcome in meningiomas, thus helping physicians in predicting which patients are more likely to experience an aggressive course of the disease.

## Conclusion

Although meningiomas are routinely recognized on hematoxylin and eosin–stained sections, sometimes their differential diagnosis with other meningeal tumors may be extremely challenging.

Evaluation of CD13 expression could be of great help in dealing with such difficult cases because (i) the combination of CD13 and EMA can potentially facilitate the diagnosis of meningioma, as all of the meningiomas tested expressed either one of the two markers or both, and (ii) none of the schwannomas nor of the SFT/HCPs showed positive immunohistochemical staining for CD13.

Moreover, loss of CD13 seems to be associated with gain of aggressive biological features, as benign and atypical meningiomas are more likely to express such marker than high-grade anaplastic meningiomas. Further studies are then required to demonstrate whether CD13 loss in meningioma could bear a prognostic relevant meaning.

## Supplementary Information

Below is the link to the electronic supplementary material.

Supplementary file1 (DOCX 45 KB)

## Abbreviations

CNS: Central nervous system
SFT/HPC: Solitary fibrous tumor/hemangiopericytoma
EMA: Epithelial membrane antigen
APN: Aminopeptidase N
