# Supplementary material for: CD13 is a useful tool in the differential diagnosis of meningiomas with potential biological and prognostic implications
Source: Virchows Arch. 2022 Feb 25;480(6):1223–30. doi: 10.1007/s00428-022-03304-9 (PMC9184408; doi:10.1007/s00428-022-03304-9)
Supplement: Supplementary file 1 — Supplementary file1 (DOCX 45 KB) [file 428_2022_3304_MOESM1_ESM.docx]

**Table S1.** Clinical and pathological characteristics of the tumors of the actual series.

| **Case** | **Age** | **Gender** | **Size (cm)** | **Site/Laterality** | **Histotype** |
| --- | --- | --- | --- | --- | --- |
| **Grade I meningiomas** |  |  |  |  |  |
| 1 | 60 | F | 4 | pontocerebellar/N.A. | meningothelial |
| 2 | 35 | M | 1,5 | sellar tubercolus/N.A. | meningothelial |
| 3 | 62 | F | 2,5 | frontal/L | meningothelial |
| 4 | 71 | M | 10 | frontal/R | meningothelial |
| 5 | 73 | F | 1,5 | parietal/R | meningothelial |
| 6 | 64 | M | 4,5 | temporal/R | meningothelial |
| 7 | 45 | F | 2,5 | petroclival/L | meningothelial |
| 8 | 31 | F | 3 | pontocerebellar/N.A. | meningothelial |
| 9 | 49 | F | 3,5 | temporal/L | secretory |
| 10 | 53 | F | 3,2 | pontocerebellar/L | meningothelial |
| 11 | 72 | F | 4 | pontocerebellar/L | fibrous |
| 12 | 63 | F | 5,5 | olfactory tract/L | meningothelial |
| 13 | 76 | M | 1,4 | thoracic spine/N.A. | psammomatous |
| 14 | 65 | F | 3,5 | sellar tubercolus/R | meningothelial |
| 15 | 57 | F | 8 | olfactory tract/N.A. | meningothelial |
| 16 | 51 | F | 5 | falx cerebri/N.A. | secretory |
| 17 | 56 | F | 3,5 | parasagittal/N.A. | meningothelial |
| 18 | 69 | F | 9 | clinoidal/R | meningothelial |
| 19 | 54 | M | 4,5 | frontoparietal/R | meningothelial |
| 20 | 73 | F | 1,5 | temporal/L | meningothelial |
| 21 | 61 | F | 3,5 | parietoccipital/R | fibrous |
| 22 | 77 | F | 4,5 | temporal/L | fibrous |
| 23 | 75 | F | 1 | thoracic spine/N.A. | meningothelial |
| 24 | 68 | M | 12 | frontoparietal/R | meningothelial |
| 25 | 44 | F | 2 | sphenoid/N.A. | fibrous |
| 26 | 74 | F | 1,5 | thoracic spine/N.A. | meningothelial |
| 27 | 44 | F | 8 | occipital/L | fibrous |
| 28 | 54 | F | 5,5 | sphenoid/N.A. | meningothelial |
| 29 | 52 | F | 4,5 | falx cerebri/N.A. | fibrous |
| 30 | 53 | F | 6 | clinoidal/L | meningothelial |
| 31 | 62 | F | 4 | sphenorbital/N.A. | meningothelial |
| 32 | 53 | F | 4,5 | sphenorbital/L | meningothelial |
| 33 | 68 | F | 2,3 | temporal/R | meningothelial |
| 34 | 18 | F | 2 | pontocerebellar/N.A. | meningothelial |
| 35 | 54 | F | 2 | cervical spine/L | meningothelial |
| 36 | 71 | F | 3 | lumbar spine/N.A. | fibrous |
| 37 | 77 | M | 3 | sphenoid/N.A. | fibrous |
| 38 | 76 | M | 2 | pontocerebellar/N.A. | fibrous |
| 39 | 65 | F | 10 | olfactory tract/N.A. | meningothelial |
| 40 | 63 | F | 1,5 | pontocerebellar/R | meningothelial |
| 41 | 79 | M | 4 | pontocerebellar/L | fibrous |
| 42 | 80 | F | 2,5 | thoracic spine/N.A. | meningothelial |
| 43 | 75 | M | 13 | temporal/N.A. | meningothelial |
| 44 | 78 | F | 3,5 | temporal/L | meningothelial |
| 45 | 47 | F | 3 | pontocerebellar/N.A. | meningothelial |
| 46 | 73 | M | 8 | olfactory tract/N.A. | meningothelial |
| 47 | 62 | M | 3 | occipital/R | meningothelial |
| 48 | 86 | F | 1,5 | thoracic spine/N.A. | meningothelial |
| 49 | 55 | F | 1,5 | clinoidal/L | meningothelial |
| 50 | 78 | M | 1,3 | cervical spine/N.A. | psammomatous |
| 51 | 49 | F | 4 | pontocerebellar/R | fibrous |
| 52 | 81 | M | 0,5 | frontoparietal/L | meningothelial |
| 53 | 51 | F | 2 | pontocerebellar/R | secretory |
| 54 | 48 | M | 4,5 | lateral ventricul/N.A. | fibrous |
| 55 | 60 | M | 3 | petroclival/R | meningothelial |
| 56 | 62 | F | 3,5 | sphenoetmoidal/N.A. | meningothelial |
| 57 | 68 | F | 2 | thoracic spine/N.A. | meningothelial |
| 58 | 71 | F | 1,5 | N.A. | meningothelial |
| 59 | 64 | F | 2,5 | thoracic spine/N.A. | meningothelial |
| 60 | 70 | F | 1 | cervical-thoracic spine/N.A. | meningothelial |
| 61 | 62 | F | 3 | frontoparietal/L | fibrous |
| 62 | 63 | M | 3 | pontocerebellar/R | fibrous |
| 63 | 45 | F | 7,5 | olfactory tract/N.A. | microcystic |
| 64 | 63 | M | 2 | cerebellar tentorium/L | fibrous |
| 65 | 45 | F | 4,5 | pontocerebellar/L | meningothelial |
| 66 | 63 | M | 4 | falx cerebri/N.A. | fibrous |
| 67 | 54 | F | 4,5 | sphenoid/N.A. | meningothelial |
| 68 | 32 | F | 3,5 | cervical spine/N.A. | meningothelial |
| 69 | 54 | F | 6,5 | parasagittal/L | meningothelial |
| 70 | 72 | F | 7,5 | frontal/N.A. | meningothelial |
| 71 | 82 | M | 3,5 | sphenoid/L | meningothelial |
| 72 | 78 | F | 10 | frontal/L | meningothelial |
| 73 | 71 | F | 1,5 | frontal/N.A. | meningothelial |
| 74 | 66 | F | 3,5 | cerebellum/R | meningothelial |
| 75 | 42 | F | 9,5 | falx cerebri/N.A. | meningothelial |
| 76 | 79 | M | 7 | cerebellum/L | fibrous |
| 77 | 63 | M | 1,5 | pontocerebellar/R | meningothelial |
| 78 | 66 | M | 5,5 | parietal/L | meningothelial |
| 79 | 54 | M | 10,5 | frontal/R | meningothelial |
| 80 | 34 | F | 6 | frontal/N.A. | meningothelial |
| 81 | 39 | F | 2,7 | optical nerve/R | meningothelial |
| 82 | 58 | F | 2 | pontocerebellar/R | secretory |
| 83 | 86 | M | 13 | frontal/R | fibrous |
| 84 | 34 | F | 3,5 | thoracic spine/N.A. | meningothelial |
| 85 | 72 | F | 5 | pontocerebellar/R | meningothelial |
| 86 | 38 | F | 11 | olfactory tract/R | meningothelial |
| 87 | 39 | F | 3 | sellar tubercolus/N.A. | meningothelial |
| 88 | 38 | F | 3 | temporoparietal/R | fibrous |
| 89 | 52 | F | 1,3 | foramen magnum/N.A. | microcystic |
| 90 | 62 | F | 4 | frontal/R | microcystic |
| 91 | 77 | F | 3 | frontal/R | secretory |
| 92 | 51 | F | 6 | falx cerebri/N.A. | fibrous |
| 93 | 72 | F | 3,5 | N.A. | meningothelial |
| 94 | 75 | F | 11,5 | frontal/R | meningothelial |
| 95 | 39 | F | 4,5 | falx cerebri/R | fibrous |
| 96 | 53 | F | 2,5 | parasagittal/R | meningothelial |
| 97 | 58 | F | 4 | olfactory tract/N.A. | meningothelial |
| 98 | 19 | F | 2,5 | optical nerve/N.A. | meningothelial |
| 99 | 59 | F | 1,5 | clivus/L | meningothelial |
| 100 | 54 | F | 3,5 | falx cerebri/L | meningothelial |
| **Grade II meningiomas** |  |  |  |  |  |
| 1 | 54 | F | 3 | cervical-thoracic spine/N.A. | meningothelial |
| 2 | 85 | M | 6 | falx cerebri/L | fibrous |
| 3 | 52 | F | 16 | frontal/L | meningothelial |
| 4 | 71 | F | 8 | N.A. | meningothelial |
| 5 | 80 | M | 4 | sphenoid/L | meningothelial |
| 6 | 16 | F | 5 | temporoparietal/R | meningothelial |
| 7 | 78 | M | 12 | frontoparietal/N.A. | meningothelial |
| 8 | 39 | M | 4,5 | frontoparietal/R | meningothelial |
| 9 | 82 | F | 5 | temporal/L | fibrous |
| 10 | 44 | F | 5,5 | frontoparietal/R | meningothelial |
| 11 | 69 | M | 1 | frontal/R | meningothelial |
| 12 | 61 | M | 11 | parietal/L | fibrous |
| 13 | 71 | M | 5 | parietal/L | meningothelial |
| 14 | 62 | M | 3 | sphenoid/N.A. | meningothelial |
| 15 | 65 | F | 14 | falx cerebri/N.A. | meningothelial |
| 16 | 70 | F | 7 | parietotemporal/R | meningothelial |
| 17 | 72 | M | 7,5 | frontal/R | meningothelial |
| 18 | 69 | M | 5 | frontal/R | meningothelial |
| 19 | 69 | M | 6,5 | parietal/R | fibrous |
| 20 | 77 | M | 8,5 | parietal/R | meningothelial |
| 21 | 62 | F | 2,5 | frontoparietal/R | meningothelial |
| 22 | 57 | F | 5 | pontocerebellar/N.A. | fibrous |
| 23 | 71 | F | 12,5 | parietocciopital/L | meningothelial |
| 24 | 76 | F | 8 | frontal/R | meningothelial |
| 25 | 68 | M | 6,5 | frontoparietal/R | meningothelial |
| 26 | 82 | M | 13 | falx cerebri/L | meningothelial |
| 27 | 74 | M | 8 | falx cerebri/N.A. | meningothelial |
| 28 | 49 | F | 4 | frontal/R | meningothelial |
| 29 | 61 | M | 3 | falx cerebri/N.A. | meningothelial |
| 30 | 76 | F | 6,5 | temporal/L | meningothelial |
| 31 | 72 | M | 5,5 | occipital/N.A. | meningothelial |
| 32 | 75 | M | 7,7 | frontal/L | meningothelial |
| 33 | 78 | M | 3,5 | olfactory tract/N.A. | meningothelial |
| 34 | 79 | M | 13 | frontal/L | meningothelial |
| 35 | 60 | M | 5 | frontal/R | meningothelial |
| 36 | 55 | F | 15 | occipital/R | meningothelial |
| 37 | 68 | M | 4,5 | parietal/N.A. | fibrous |
| 38 | 66 | M | 13 | parietal/L | fibrous |
| 39 | 63 | M | 3 | frontal/L | fibrous |
| 40 | 80 | F | 6 | frontal/L | meningothelial |
| 41 | 64 | F | 5,5 | parietal/R | meningothelial |
| 42 | 70 | M | 11 | parietal/R | meningothelial |
| 43 | 37 | F | 2,5 | falx cerebri/L | fibrous |
| 44 | 66 | F | 5 | sphenoid/N.A. | meningothelial |
| 45 | 70 | M | 4 | temporal/N.A. | fibrous |
| 46 | 70 | F | 11 | parietal/N.A. | meningothelial |
| 47 | 71 | F | 5 | falx cerebri/L | meningothelial |
| 48 | 48 | F | 12 | frontal/L | meningothelial |
| 49 | 65 | M | 8 | frontal/R | meningothelial |
| 50 | 48 | F | 4 | frontal/L | clear cell |
| 51 | 77 | F | 5 | falx cerebri/R | meningothelial |
| 52 | 54 | M | 7 | falx cerebri/N.A. | meningothelial |
| 53 | 57 | M | 5 | parietal/R | meningothelial |
| 54 | 74 | M | 4,5 | olfactory tract/N.A. | meningothelial |
| 55 | 38 | M | 2 | petroclival/R | meningothelial |
| 56 | 59 | M | 7,5 | olfactory tract/N.A. | meningothelial |
| 57 | 69 | M | 6 | cerebellum/N.A. | meningothelial |
| 58 | 66 | M | 2,5 | temporal/R | meningothelial |
| 59 | 72 | M | 13 | temporal/R | meningothelial |
| 60 | 58 | M | 16 | frontoparietal/L | meningothelial |
| 61 | 68 | M | 8 | temporal/R | meningothelial |
| 62 | 73 | F | 4 | sphenoid/N.A. | meningothelial |
| 63 | 57 | F | 3,7 | temporal/R | meningothelial |
| 64 | 57 | F | 3,5 | frontal/N.A. | meningothelial |
| 65 | 68 | M | 6 | frontal/L | meningothelial |
| 66 | 44 | M | 5 | lateral ventricle/N.A. | meningothelial |
| 67 | 80 | M | 3 | falx cerebri/L | meningothelial |
| 68 | 78 | M | 3,2 | falx cerebri/R | meningothelial |
| 69 | 63 | F | 2,5 | parietal/R | fibrous |
| 70 | 63 | F | 5 | falx cerebri/N.A. | meningothelial |
| 71 | 81 | F | 2,7 | falx cerebri/N.A. | fibrous |
| 72 | 58 | F | 13 | sphenoid/L | meningothelial |
| 73 | 41 | M | 3,5 | parietal/R | meningothelial |
| 74 | 42 | F | 8 | temporal/R | meningothelial |
| 75 | 50 | F | 6,5 | parietal/L | meningothelial |
| 76 | 59 | M | 15,5 | frontal/R | meningothelial |
| 77 | 82 | F | 3,5 | frontal/L | fibrous |
| 78 | 82 | M | 7 | cerebellum/R | meningothelial |
| 77 | 66 | F | 6 | falx cerebri/N.A. | meningothelial |
| 80 | 69 | F | 6,5 | frontoparietal/L | meningothelial |
| 81 | 63 | F | 10 | occipital/R | meningothelial |
| 82 | 78 | M | 3 | falx cerebri/L | meningothelial |
| 83 | 64 | M | 3,5 | N.A. | meningothelial |
| 84 | 61 | F | 5,5 | frontal/L | meningothelial |
| 85 | 68 | M | 3 | temporal/L | meningothelial |
| 86 | 55 | M | 5,5 | temporal/L | meningothelial |
| 87 | 74 | M | 2 | petroclival/L | meningothelial |
| 88 | 80 | M | 7,5 | N.A. | meningothelial |
| 89 | 57 | M | 16 | temporal/R | meningothelial |
| 90 | 37 | F | 5,5 | parietotemporal/R | fibrous |
| 91 | 73 | M | 5,5 | parietal/L | meningothelial |
| 92 | 65 | F | 2 | pontocerebellar/L | meningothelial |
| 93 | 57 | F | 10 | temporal/L | meningothelial |
| 94 | 23 | F | 15 | frontal/L | meningothelial |
| 95 | 71 | F | 11,5 | parietocciopital/L | meningothelial |
| 96 | 79 | F | 8,5 | parietotemporal/R | meningothelial |
| 97 | 50 | F | 7,5 | N.A. | meningothelial |
| 98 | 76 | M | 10,5 | olfactory tract/N.A. | meningothelial |
| 99 | 62 | F | 4,5 | olfactory tract/N.A. | meningothelial |
| 100 | 53 | F | 6 | frontal/L | meningothelial |
| **Grade III meningiomas** |  |  |  |  |  |
| 1 | 75 | M | 3 | brain/N.A. | meningothelial |
| 2 | 53 | M | 8 | falx cerebri/N.A. | meningothelial |
| 3 | 45 | M | 2 | temporal/L | meningothelial |
| 4 | 22 | F | 4,5 | parietal/L | meningothelial |
| 5 | 74 | M | 7,5 | frontal/R | meningothelial |
| 6 | 64 | M | 2,5 | frontal/L | meningothelial |
| 7 | 22 | M | 3 | falx cerebri/N.A. | meningothelial |
| 8 | 63 | M | 2,5 | frontal/L | meningothelial |
| 9 | 59 | M | 6,5 | falx cerebri/N.A. | meningothelial |
| 10 | 56 | M | 3,5 | frontal/R | meningothelial |
| 11 | 57 | M | 3 | falx cerebri/R | meningothelial |
| 12 | 57 | M | 4 | scalp/N.A. | meningothelial |
| 13 | 56 | M | 9 | frontoparietal/N.A. | meningothelial |
| 14 | 58 | M | 6 | frontal/N.A. | meningothelial |
| 15 | 38 | M | 3,5 | falx cerebri/N.A. | meningothelial |
| 16 | 71 | F | 8 | temporal/L | meningothelial |
| 17 | 71 | M | 5,5 | foramen magnum/L | meningothelial |
| 18 | 64 | M | 14 | falx cerebri/N.A. | meningothelial |
| 19 | 69 | M | 3 | occipital/R | meningothelial |
| 20 | 56 | F | 3,5 | cerebellum/L | meningothelial |
| 21 | 80 | F | 7 | retroauricolar/L | meningothelial |
| 22 | 50 | M | 6 | parietal/R | meningothelial |
| 23 | 59 | M | 4,5 | parietotemporal/R | meningothelial |
| 24 | 62 | F | 4 | falx cerebri/L | meningothelial |
| 25 | 79 | F | 2 | tentorium cerebelli/N.A. | meningothelial |
| **Schwannomas** |  |  |  |  |  |
| 1 | 61 | M | 2 | pontocerebellar/L | N.A. |
| 2 | 67 | F | 2,5 | pontocerebellar/L | N.A. |
| 3 | 65 | M | 1,5 | cervical spine/N.A. | N.A. |
| 4 | 71 | F | 2,5 | cervical spine/N.A. | N.A. |
| 5 | 64 | F | 2,5 | pontocerebellar/L | N.A. |
| 6 | 53 | M | 1,5 | pontocerebellar/L | N.A. |
| 7 | 59 | M | 1,5 | pontocerebellar/R | N.A. |
| 8 | 44 | F | 1,2 | pontocerebellar/R | N.A. |
| 9 | 66 | M | 1,3 | pontocerebellar/R | N.A. |
| 10 | 64 | M | 0,7 | pontocerebellar/L | N.A. |
| 11 | 52 | M | 2 | pontocerebellar/N.A. | N.A. |
| 12 | 54 | M | 2 | lombar spine/N.A. | N.A. |
| 13 | 68 | M | 2,5 | lombar spine/N.A. | N.A. |
| 14 | 22 | M | 3 | lombar spine/N.A. | N.A. |
| 15 | 50 | F | 2,5 | pontocerebellar/L | N.A. |
| **TFS/Eps** |  |  |  |  |  |
| 1 | 58 | F | 3 | falx cerebri/N.A. | N.A. |
| 2 | 47 | M | 2,3 | falx cerebri/L | N.A. |
| 3 | 69 | M | 5,2 | cervical spine/N.A. | N.A. |
| 4 | 82 | F | 2,8 | skull base/N.A. | N.A. |
| 5 | 62 | M | 4 | lombar spine/N.A. | N.A. |
| 6 | 86 | F | 7,5 | falx cerebri/N.A. | N.A. |
| 7 | 40 | M | 9,5 | occipital/R | N.A. |
| 8 | 77 | M | 1,8 | thoracic spine/N.A. | N.A. |
| 9 | 45 | M | 5 | falx cerebri/L | N.A. |
| 10 | 68 | M | 8 | spinal/L | N.A. |
| 11 | 57 | M | 7 | skull base/N.A. | N.A. |
| 12 | 70 | F | 5,5 | falx cerebri/R | N.A. |
| 13 | 80 | F | 6,5 | skull base/L | N.A. |
| 14 | 66 | M | 2 | cervical-thoracic spine/N.A. | N.A. |
| 15 | 61 | M | 8 | dorsal-lombar spine/N.A. | N.A. |
| 16 | 78 | M | 4,5 | skull base/R | N.A. |
| 17 | 80 | F | 6 | falx cerebri/N.A. | N.A. |
| 18 | 35 | F | 2 | sphenoid/R | N.A. |
| 19 | 68 | F | 5 | skull base/N.A. | N.A. |
| 20 | 33 | M | 2,3 | falx cerebri/R | N.A. |

Abbreviations: F: female, M: male, R: right, L: left, SFT/HPC: solitary fibrous tumor/hemangiopericytoma, N.A.: not available.
